# Translational simulation: not ‘where?’ but ‘why?’ A functional view of in situ simulation

**DOI:** 10.1186/s41077-017-0052-3

**Published:** 2017-10-19

**Authors:** Victoria Brazil

**Affiliations:** Faculty of Health Sciences and Medicine, Bond University, Gold Coast, Australia

**Keywords:** In situ simulation, Healthcare simulation, Quality improvement, Translational science

## Abstract

Healthcare simulation has been widely adopted for health professional education at all stages of training and practice and across cognitive, procedural, communication and teamwork domains. Recent enthusiasm for in situ simulation—delivered in the real clinical environment—cites improved transfer of knowledge and skills into real-world practice, as well as opportunities to identify latent safety threats and other workplace-specific issues.

However, describing simulation type according to place may not be helpful. Instead, I propose the term translational simulation as a functional term for how simulation may be connected directly with health service priorities and patient outcomes, through interventional and diagnostic functions, independent of the location of the simulation activity.

**Electronic supplementary material:**

The online version of this article (10.1186/s41077-017-0052-3) contains supplementary material, which is available to authorized users.

## Background

Healthcare simulation has been widely adopted for health professional education at all stages of training and practice and across cognitive, procedural, communication and teamwork domains. Descriptions of simulation type often use the device (e.g. high fidelity mannequin simulation) or place, e.g. in situ simulation (ISS), but these descriptors underplay the critical importance of outcomes from simulation, e.g. individual competence, team behaviours or patient level outcomes. In this article, I argue for using the *function* of simulation as its descriptor and offer *translational simulation* as an appropriate term for describing the subset of simulation activities that are directly focused on improving healthcare processes and outcomes.

## Current descriptors for simulation can be problematic

Gaba’s classic description of ‘dimensions’ [1] underline the diversity of simulation modalities and applications. Commonly used terms like ‘ínterprofessional’, ‘high fidelity’, ‘centre based’ or ‘Rapid Cycle Deliberate Practice sim’ focus on the process by which the simulation is conducted, through a lens of provider or participant. These descriptors are useful in matching our process to our aims. However, Gaba’s dimension 1—the purpose and aims of the simulation—risks conceptual and practical neglect if we become too focused on those processes.

The emergence of in situ simulation (ISS)—conducted in the real clinical environment—offers an example. There are benefits to simulation training conducted in specialized simulation environments or centres—trained faculty and advanced training equipment can be concentrated in one location, and the dedicated space allows the participants to concentrate on the learning tasks. Across all domains of practice, there are barriers to transfer of the knowledge and skills acquired in the simulation centre into real-world practice, including ad hoc teams, unfamiliar equipment and environments, institutional policies and procedures, health service culture and departmental tribalism [2, 3]. Simulation training conducted in situ provides opportunities to address these barriers [4–7].

The emergence of training in the clinical environment (and its powerful label as ‘ISS’) has led to unhelpful debate regarding whether this approach or centre-based simulation is superior [8], with all the attendant challenges in defining superiority, including cost and feasibility [9]. At worst, the labelling by location reduces the impact, as providers lose clarity about specific aims because of the assumed general superiority of ISS.

The task of the healthcare simulation provider is to match the modality to the purpose required. We need a functional descriptor for those subsets of simulation activities that are connected *directly* with health service priorities and patient outcomes, through therapeutic and diagnostic functions, and independent of the location of the simulation activity.

This article offers support for a terminology change and conceptual shift through examples from the literature and my own experience with in situ simulation programs at two institutions. Drawing on language from the biosciences context, explicit reference to *translational simulation* may advance the field by sharpening the focus on patient and systems outcomes.

## But isn't all simulation about improving outcomes for patients?

The training of individuals and teams in communication, technical skills and teamwork is *necessary* for improved patient safety and outcomes, but not *sufficient*. Assumptions about how closely linked any educational activities are with patient level outcomes should be questioned but are hard to test. Efforts to rank healthcare simulation activities which offer most direct outcome benefit are helpful for directing educational and research focus [10, 11]. These authors also suggest ‘system probing’ [11], ‘evaluation of microsystems’ [12] and ‘implementation science’ [13] as critical adjuncts to individual and team training and recognize that many patient outcome level improvements involve ‘complex service interventions’ [14].

Translational simulation activities encompass modalities, locations and delivery methods that address that gap.

## The translational simulation concept (Fig. 1)

Berwick’s landmark paper provides a framework for how we improve care through system approaches [15]. In developing the Plan Do Study Act cycle, he asks three questions that provide a framework for how simulation might be used for healthcare improvement.

**Fig. 1:**
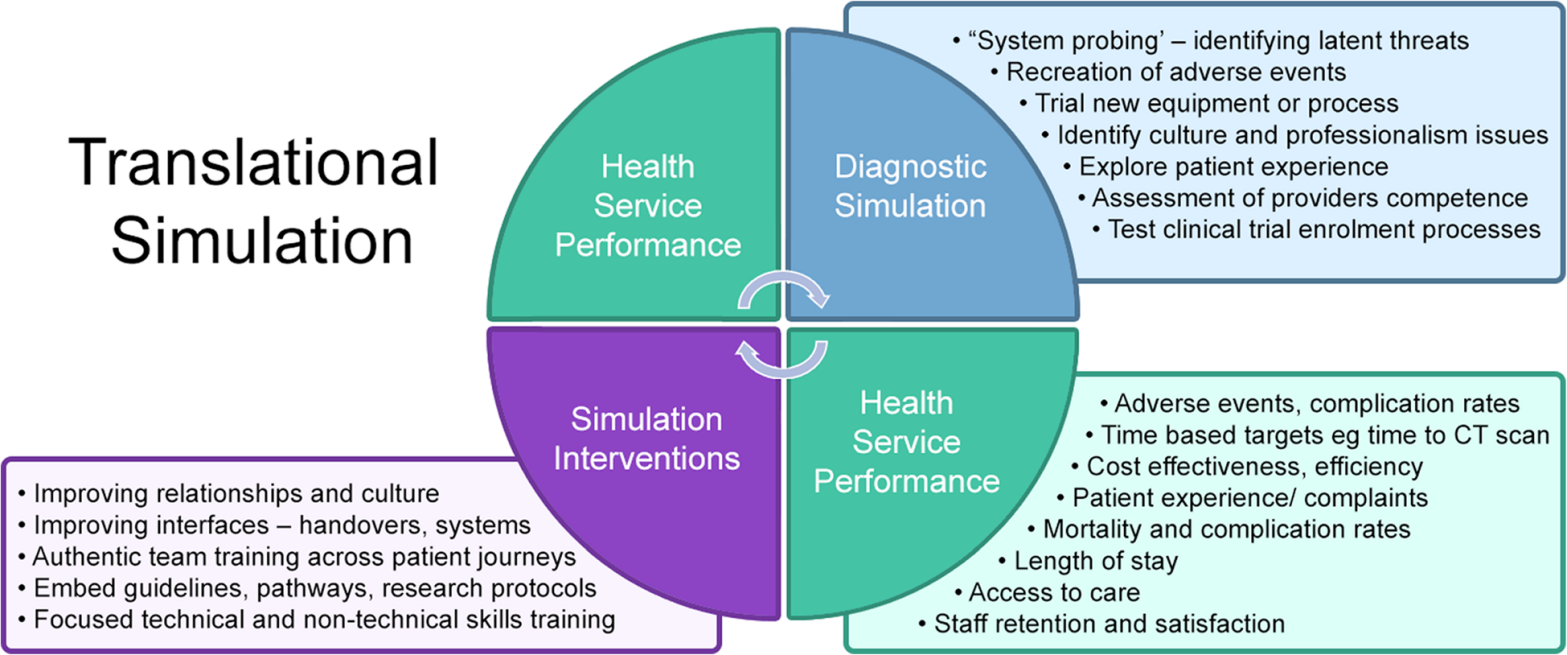
Translational simulation

*1. What are we trying to accomplish?* Healthcare simulation activities often aim for too much. Improving technical skills and testing the communication systems in a hospital are both worthy objectives, but the chances of either being achieved decreases if we try and focus on both.

*2. How will I know if a change leads to an improvement?* Translational simulation can be part of the *diagnostics* in health systems—identifying issues preventing excellent care and assessing outcomes achieved in response to interventions.

*3. What changes could we make that we think will result in improvement*? Appropriately targeted, translational simulation offers a range of *interventions* for individual, team and system level improvement, ideally embedded within an integrated ‘translational science’ model [13].

## Effective translational simulation *interventions* for health service institutions

In the context of translational simulation, *education and training* is directed at a specific healthcare outcome target, not just an assumption of improved system performance as a result of improved individual knowledge or skills.

Frequently cited examples include lowered infection rates as a result of targeted training for central line insertion [16], reduction in perinatal asphyxia and neonatal hypoxic-ischaemic encephalopathy (HIE) following team training for obstetric emergencies [17] and survival in cardiac arrest with a rapid cycle deliberate practice simulation approach [18]. Improved outcomes for trauma patients, including decreased times from patient arrival to the CT scanner and to the operating room, have been reported following an in situ TeamSTEPPs educational intervention [19]. All of these targets involve individual and team knowledge and skills, but also complex, context-specific system issues. These translational simulation activities are likely to be most effective when explicitly integrated with an institutional quality improvement program [20].

Targeted translational simulation interventions may also be designed to *embed a specific process or procedure*. Examples include *red blanket* protocols [21] (for rapid transfer of critically hypotensive trauma patients to the operating theatre), massive transfusion protocols, endotracheal intubation checklists [22], or to practice using new equipment. The design of these simulations will be focused on systems and processes, rather than individual or team knowledge and skills. The simulation participants are variables held more or less constant to focus on the process or system. While there is still an individual learning impact, the primary purpose of the activity is translational.

Targeting *improved team culture and professionalism* is less frequent, although often a secondary outcome of translational simulation activities. The extent to which improvements in this less tangible, behavioral target contribute to improvements in performance is hard to quantify, although rudeness has been correlated with decreased team performance in simulations [23].

Despite the enthusiasm for in situ delivery, effective translational simulation activities may be conducted in traditional simulation centres if the healthcare target can be achieved through training in that environment. Translational simulation does not imply a place, but rather a function. The site should be determined by the functional task alignment [24] of environment and team composition for the improvement target.

## *Diagnostic* translational simulation in health service institutions

Early in situ simulation programs focusing primarily on educational outcomes found serendipitous benefits in identifying problems in the environment and systems in which the individuals or teams were training [25]. Subsequently, programs have been specifically designed to test health service performance, such as the identification of latent threats in trauma and in paediatric critical care [26], or to understand weaknesses in processes such as blood transfusion [27]. These programs commonly reveal issues related to equipment, communication and institutional procedures [28, 29].

Translational simulation offers diagnostic opportunities when preparing for the *opening of new departments* or health service facilities [30], allowing redesign of equipment, layout or workflows [31] for maximal efficiency and safety. As a complement to existing international standards for testing of medical device design [32], translational simulation activities are the next step in assessing the equipment’s utility with the local human factors and institutional context.

Less well articulated is the role of translational simulation activities, especially those conducted across departmental interfaces, in *diagnosing cultural and professionalism issues*. These issues can critically influence the delivery of safe and effective healthcare and may be obvious to in situ simulation debriefers but are not easily measured.

*Assessment of individual providers’ readiness for practice*, or continuing competency, may have an important translational impact. With improvements in physical resemblance, environmental fidelity and scenario functional task alignment, simulation has achieved greater acceptability for high-stakes assessment of students and practicing providers. This is most likely to be valid (and hence linked to patient outcomes) if closely linked to the actual tasks and context in which performance will be required, e.g. a final year medical student OSCE is less likely to have a translational impact than a lumbar puncture performed in simulation just before it is required on a real patient [33].

## How do we know if translational simulation has a translational impact?

Improved performance is easier to measure in areas such as procedural skills and validated teamwork scales, which may or may not have a translational impact. McGaghie and colleagues have led an emerging simulation research theme providing guidance for measuring outcomes at the ‘T3’ level, i.e. patient outcomes and institutional or system level [13]. Translational simulation would be viewed as a ‘complex service intervention’ within this framework and includes multifaceted evaluation of quality outcomes—efficiency, effectiveness, safety, patient centredness and equity. Formal integration of translational simulation within an institutional quality improvement framework and governance is likely to support disciplined measurement of outcomes.

Translational simulation research may be enhanced if it ‘joins the conversation’ [34] with quality improvement scholars. Reporting guidelines for healthcare simulation research [35] are excellent but modality-focused. Researchers might also consider guidelines specifically designed for quality improvement [36] and aligned publication vehicles.

## A policy approach to translational simulation? (Fig. 2)

Many described interventional and diagnostic translational simulation activities are effective at a specific departmental or ‘frontline’ level, or for a specific patient journey. Consistent with lessons from quality improvement, Plan Do Study Act (PDSA) cycles are most effective the closer they are to the clinical teams and their work.

**Fig. 2:**
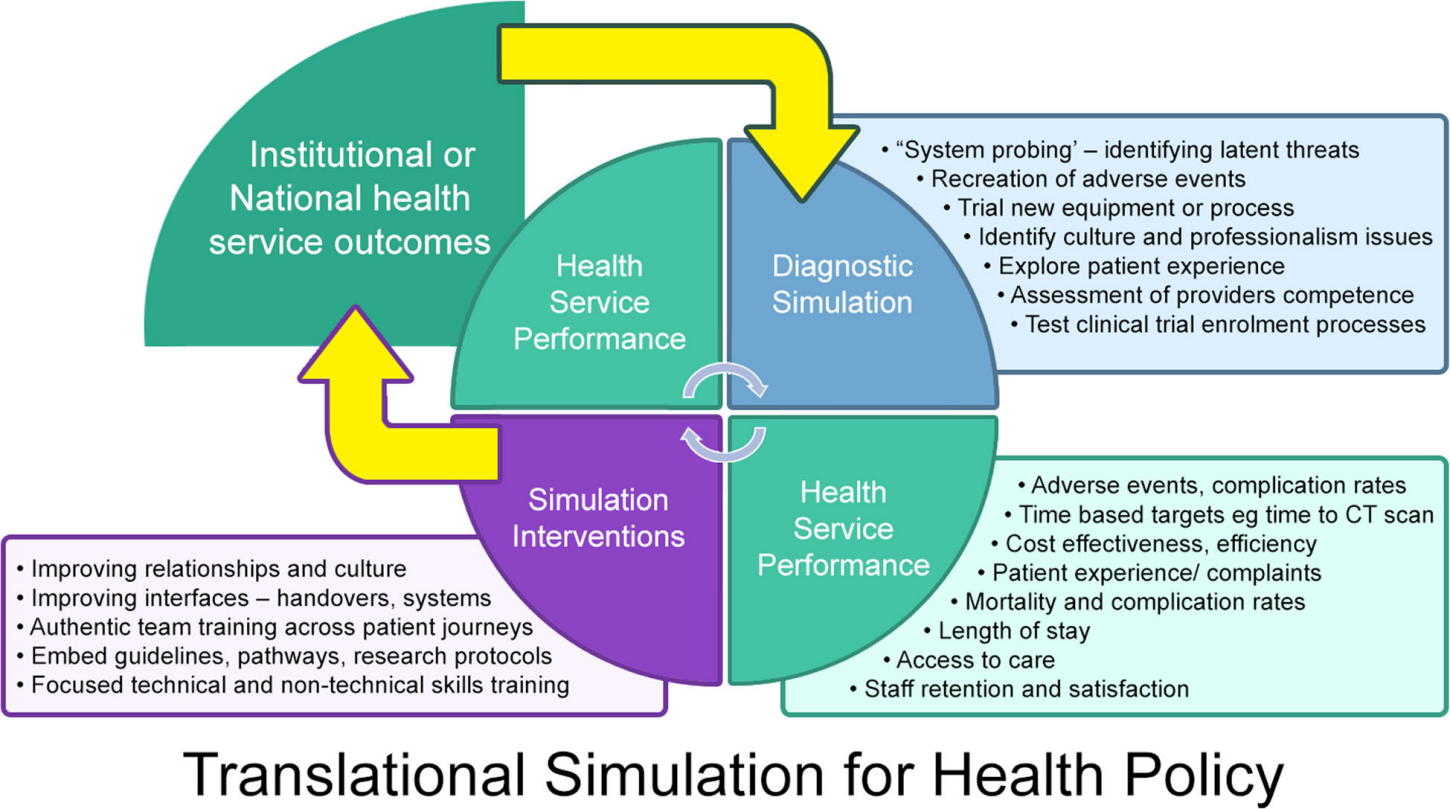
Translational simulation for health policy

However, translational simulation also needs to align with organizational and health policy level priorities to be most effective. Targets for interventional translational simulation have to be those that matter to patients and health services. Formal processes are needed for conveying diagnostic information about healthcare processes and system function from translational simulation activities to those who review performance at an organizational and national level.

Translational simulation encompasses the concept of *Systems Integration*—defined by the Society for Simulation in Healthcare (SSH) as ‘those simulation programs which demonstrate consistent, planned, collaborative, integrated and iterative application of simulation-based assessment and teaching activities with systems engineering and risk management principles to achieve excellent bedside clinical care, enhanced patient safety, and improved metrics across the healthcare system’ [37]. The SSH accreditation standards emphasize governance structures and reporting relationships as evidence of translational impact. For example, ‘…clear evidence of participation by Simulation Program leadership in the design and processes of quality management system improvement activities at the organizational level.’ [37].

Additional file 1 provides a case study example of interdepartmental translational simulation at Gold Coast University hospital. Additional file 2 offers a Simulation Report Form example, used in the case study institution.

## Conclusion

The term *translational simulation* describes healthcare simulation focused directly on improving patient care and healthcare systems, through *diagnosing* safety and performance issues and delivering simulation-based *intervention*, irrespective of the location, modality or content of the simulation. It offers a functional alignment with quality improvement activities in healthcare institutions, while encompassing those educational interventions targeting practice behavior or patient outcomes. Translational simulation requires close relationships with clinical governance and quality improvement services in healthcare institutions.

A change in terminology, with an attendant clarity of focus, offers an exponential impact for healthcare simulation to be used effectively as part of comprehensive health service improvement strategies.

## Additional files

Additional file 1: Case study - Interdepartmental translational simulation at Gold Coast University Hospital (DOCX 15 kb)

Additional file 2: Simulation report form (PDF 33 kb)
