# Supplementary material for: Translational simulation: not ‘where?’ but ‘why?’ A functional view of in situ simulation
Source: Adv Simul (Lond). 2017 Oct 19;2:20. doi: 10.1186/s41077-017-0052-3 (PMC5806247; doi:10.1186/s41077-017-0052-3)
Supplement: Supplementary file 1 — Case study - Interdepartmental translational simulation at Gold Coast University Hospital (DOCX 15 kb) [file 41077_2017_52_MOESM1_ESM.docx]

**A case study example –translational simulation of patient journeys at Gold Coast University hospital**

***Setting***

The Gold Coast University Hospital(GCUH) is an 850 bed tertiary referral hospital in Queensland, Australia. The service profile includes all major subspecialties. The hospital was opened in 2013, concurrent with the establishment of the Simulation Service and a number of other new dedicated services such as Trauma, Stroke and Childrens Critical Care.

The Simulation Service is staffed by 3.5 full time equivalent (FTE) simulation educators, together with a Manager, Medical Director and part time Simulation Fellow. The activities of the Service include Basic life support, ACLS, other team training, technical and educational consultation for individual departments providing simulation, faculty development and research.

***Translational simulation activities – interventional and diagnostic***

Over the last 3 years, the service has provided monthly in situ simulations with the target of reviewing and improving the complex, time critical trauma and acute care processes. A number of targets were identified for the ‘intervention’ of translational simulation – time to CT Scan, improved use of personal protective equipment (PPE) and lead gowns, and improved professional interfaces with other departments. As the program evolved, it became clear that each simulation and debrief yielded a significant number of identified diagnostic issues, often related to equipment, communication processes, handovers and decision-making. This resulted in the adaption of a ‘sim report form’ used in another institution (pers comm J. Spur 2015) for the purposes of recording these issues and reporting them to service leads after the event. (see Additional file 1)

Other services in the hospital – anaesthesia, maternity, mental health and stroke - also requested similar simulation based exercises focused on improving interdepartmental patient journeys, and these were undertaken in a less rigid schedule.

A review of 12 months of Simulation Reports was undertaken after formal ethics waiver by the Gold Coast Hospital and Health Service Human Research Ethics Committee (reference HREC/16/QGC/185).

Of the 12 simulations reported most were focused on trauma (9) with others related to obstetric emergencies (1), acute stroke (1), and delivery of Electroconvulsive Therapy (ECT) in mental health (1).

More than ten services were involved, including pre-hospital, emergency department, operating theatre, anaesthesia, mental health, hospital security, obstetrics and various surgical subspecialties. The session were all interprofessional – doctors, nurses, social workers, radiographers and porterage staff. The high complexity of patient journeys simulated was further underlined by the large numbers of staff involved – ranging from 15 to more than 35.

Content analysis of the report forms was undertaken, within each category of the form. Brief ‘diagnostic’ examples include:-

*“Confusion over the term ‘cric kit’ – requested by anaesthetic consultant who really simply meant ‘surgical airway kit’. Melker kit then provided not preferred pre-made ‘scalpel bougie’ kit in 2nd drawer airway trolley”* Maxillofacial trauma sim

*“ED - Security staff emphasised risks to personnel and infrastructure with this kind of patient (largely from outside individuals who may attempt access) – clinical staff often don’t act with these risks in mind (eg going out to ambulance bay)”* GSW Trauma – police shooting

*“Crowd Control – Issues with large numbers of people without an active role going up to theatre. ?need for clear delineation of essential staff required for transfer.”* Paediatric trauma

*“Poor sound proofing between waiting area and ECT room – frightening for patients No private check in area for RN, psychiatrist or anaesthetist to conduct interview prior to procedure*” New ECT facility

***Future directions***

The therapeutic target of improving departmental interfaces became an increasing larger focus for these simulations, and work has commenced on a project to use Relational Coordination[1] as an outcome measure of simulation interventions, in addition to the clinical targets.

Our diagnostic findings have led to developing closer governance and reporting links between the Simulation Service and our quality improvement structures within the Gold Coast Health Service. Specifically, we are developing a module within our adverse event reporting system to allow reporting of any incidents in simulation activities (eg drug errors, equipment issues, staff or patient safety) in a similar way to incidents in the clinical environment.

1. Gittell JH, Godfrey M, Thistlethwaite J. **Interprofessional collaborative practice and relational coordination: improving healthcare through relationships**. *J Interprof Care.* 2013, 27(3):210-3.
