# Supplementary material for: Translational simulation: not ‘where?’ but ‘why?’ A functional view of in situ simulation
Source: Adv Simul (Lond). 2017 Oct 19;2:20. doi: 10.1186/s41077-017-0052-3 (PMC5806247; doi:10.1186/s41077-017-0052-3)
Supplement: Supplementary file 2 — Simulation report form (PDF 33 kb) [file 41077_2017_52_MOESM2_ESM.pdf]

Queensland Government

Gold Coast Health
Simulation Report

Type of simulation: ☐ Simulation lab ☐ In-situ

Location: **Date:**

Scenario name:

Health service outcome target:

Scenario overview:

Additional information:

Participants:

Simulation faculty:

Issues identified in session and in debrief:

Safety and environment:

Performance *(against benchmarks/targets):*

Communication/interfaces:

Knowledge:

Adapted from Metro North Hospital and Health Service.

Further reflections:

Overall:

Strengths:

Weaknesses:

Opportunities:

Issues identified in simulation delivery:

Report provided to:
